# Navigating toward standardization: A systematic review mapping outcome measures in masculinizing genital gender affirming surgery

**DOI:** 10.1080/26895269.2024.2429490

**Published:** 2024-11-20

**Authors:** Philippine J. Roijer, Marleen S. Vallinga, Melle M. F. Jorna, Thomas E. Pidgeon, Johannes C. F. Ket, Mark-Bram Bouman, Margriet G. Mullender

**Affiliations:** a Department of Plastic, Reconstructive and Hand Surgery, Amsterdam UMC, location VUmc, Amsterdam, the Netherlands; b Amsterdam Public Health Institute, Amsterdam, the Netherlands; c Department of Plastic Surgery, University Hospital Birmingham, Birmingham, UK; d Medical Library, Vrije Universiteit Amsterdam, Amsterdam, The Netherlands

**Keywords:** Gender diverse, masculinizing genital gender affirming surgery, outcome reporting, transgender

## Abstract

***Background:*** Worldwide, many different surgical procedures and techniques are being used for masculinizing genital gender affirming surgery (gGAS) in transgender and gender diverse (TGD) individuals. Studies reporting on masculinizing gGAS measure and report different outcomes, hindering comparison and thus evidenced-based treatment decision making. This study aims to systematically assess reporting heterogeneity in masculinizing gGAS studies to date.***Methods:*** A systematic review in compliance with the Cochrane Handbook for Systematic Reviews of Interventions was conducted. A multi-database literature search was performed up to September 2023. Clinical studies reporting on outcomes after masculinizing gGAS (i.e. phalloplasty, scrotoplasty, metoidioplasty and coronaplasty) in TGD individuals were included. Data extraction included reported outcomes, their definitions, timing of assessment, measurement instruments used, and primary outcomes for risk of bias evaluation.***Results:*** After screening 922 studies, 87 were included for data extraction. In total, 2077 individual (clinician- and patient reported) outcomes were reported, of which 1026 (49%) were defined. The provided definitions were inconsistent, and the degree of outcome specificity varied. Among the most reported outcomes were neo-urethral fistula (*n* = 104) and stricture (*n* = 81), partial (*n* = 56) and complete (*n* = 50) flap necrosis, hematoma (*n* = 36) and, tactile sensitivity in the neo-phallus (*n* = 36). Measurement instruments were stated for 987 (48%) outcomes, primarily using ad hoc questionnaires. The timing of outcome assessment was specified for 1116 (54%) outcomes, often described as follow-up periods ranging in duration. The primary outcome was declared in 36 (41%) studies.***Conclusion:*** This review demonstrates an extensive use of ambiguous definitions, measurement tools and assessment times following masculinizing gGAS and highlights the necessity for standardization. Adoption of an agreed-upon Core Outcome Set (COS) for masculinizing gGAS could improve the comparability and reliability of research findings and ultimately improve the treatment decision-making process.

## Introduction

Gender affirming medical care (e.g. psychological, hormonal, and surgical treatment) has generally been shown to enhance the well-being and quality of life of transgender and gender diverse (TGD) individuals (Javier et al., 2022; van de Grift et al., 2018). As part of the gender affirming treatment, some individuals (assigned female at birth) wish to undergo masculinizing genital gender affirming surgery (gGAS). Various options for masculinizing gGAS are available; including the creation of a neo-phallus (with- or without urethral lengthening), a neo-scrotum and a neo-corona (Frey et al., 2017; Lane et al., 2018).

Phalloplasty allows for the creation of a neo-phallus, using sizable autologous skin flaps. Many variations of phalloplasty techniques exist, using a variety of skin flaps harvested from a range of different donor sites on the body (Al-Tamimi, Pigot, Elfering, et al., 2020; Boczar et al., 2021; Wang et al., 2022). By contrast, the metoidioplasty procedure can be employed to construct a neo-phallus, using local tissue (Djordjevic, Stojanovic, & Bizic, 2019; Morrison et al., 2022). For each of these procedures, surgeons typically select their preferred surgical technique based on their training, experience, and the TGD individual’s anatomical considerations. There are many different techniques of masculinizing gGAS to choose from, resulting in a complex clinical decision-making process (Butcher et al., 2023).

Comprehensive knowledge about clinical outcomes—which surgical techniques give the best clinical outcomes depending on personal characteristics –, and effectiveness—what are the patient’s reported outcomes with regard to the most important objectives for them—is paramount to enable decision-making about the treatment and select the best fitting treatment option for each individual. Although the literature on this topic is expanding rapidly, it currently does not allow for comparison between the results of different surgical procedures and techniques (Morrison et al., 2016). In addition, very little is known about the effectiveness of masculinizing gGAS as assessed from a TGD individual’s perspective.

To advance knowledge and enable evidence-based clinical decision-making, standardizing outcome reporting in masculinizing gGAS research is imperative (Wang et al., 2022). To this end, a Core Outcome Set (COS) is currently under development, consisting of uniformly defined outcomes that should be consistently measured and reported in all clinical trials relating to masculinizing gGAS (Roijer et al., 2023). This systematic review represents an initial step in this development, offering a comprehensive overview of outcomes reported following masculinizing gGAS procedures in TGD individuals. We will identify all outcomes, their reported definitions, measurement instruments used, and the timing of assessments reported following masculinizing gGAS. Furthermore, we aim to assess the proportion of studies declaring a primary outcome.

## Methods

A systematic review was conducted of all relevant literature on outcomes after masculinizing gGAS procedures performed in TGD diverse individuals. The review was performed in compliance with the Cochrane Handbook (Chandler et al., 2023) and reported according to the Preferred Reporting Items for Systematic Review and Meta-Analysis (PRISMA) statement (Page et al., 2021) and the Core Outcome Measures in Effectiveness Trials (COMET) Handbook (Williamson et al., 2017). The protocol is registered in the International Prospective Register of Systematic Reviews (PROSPERO 2022 CRD42022347400) (Roijer et al., 2022). The methods used in this systematic review closely follow those of a parallel review on outcome measures in feminizing genital gender-affirming surgery (Pidgeon et al., 2023). Both reviews share similar objectives and are part of The GenderCOS project (Roijer et al., 2023), which develops a COS for both feminizing and masculinizing genital gender-affirming surgery.

### Search strategy

The search strategy was developed in collaboration with a medical information specialist. The search was conducted with controlled terms and free text terms for synonyms of ‘phalloplasty’, ‘metoidioplasty’, ‘scrotoplasty’ and ‘coronaplasty’ combined with synonyms of ‘transgender persons’ or ‘gender diversity’. The search was performed without restrictions for methodology, date or language in September 2023. Electronic databases that were searched included: OVID/MEDLINE, Embase.com, Clarivate Analytics/Web of Science Core Collection, Elsevier/Scopus, EBSCO/APA PsycINFO and Proquest/International Bibliography of Social Sciences (IBSS). Additionally Google Scholar. Duplicate articles were excluded using Endnote X20.0.1 (Clarivate^™^). The full search strategy can be found in the supplementary material 1.

### Study inclusion criteria

All published clinical studies with full text available in English, which meet the PICO criteria described below, were eligible for inclusion. *Population:* live human subjects, >90% of study population were TGD individuals undergoing genital masculinizing surgery for gender affirmation. There were no age limits. *Intervention and comparators*: genital gender-affirming reconstructive phalloplasty, scrotoplasty, metoidioplasty and coronaplasty (and comparators if applicable), as either primary or revision/secondary procedure, with- or without urethral lengthening. *Outcome*: any outcome reported after the previously stated interventions.

### Study exclusion criteria

All secondary research such as systematic reviews and meta-analyses were excluded. Case reports, non-research articles, conference proceedings, abstracts alone, and animal/cadaveric studies were excluded. Studies examining penile reconstruction for patients with disorders of sexual development, or within cisgender men were not considered. Studies reporting on non-surgical interventions relating to masculinizing genital surgery e.g. esthetic techniques, were excluded.

### Study screening

Study screening (based on title and abstract) and selection (based on full text review) was independently done using Rayyan software (Ouzzani et al., 2016) by two reviewers (PJR, MSV) who cross-checked their results. Reasons for exclusion were recorded. Any disagreement was resolved between reviewers and followed by senior researcher arbitration if necessary. Inter-rater consistency was assessed using the Kappa-score (Cohen, 1960).

### Data extraction process

Data extraction was primarily done by one reviewer (PJR) using Microsoft Excel (Microsoft Corporation, 2018). To ensure extraction accuracy and consistency, data from the first 10% of included articles were also independently extracted by a second reviewer (MMFJ). Any discrepancies were discussed and resolved. Dual data extraction from additional articles after the initial 10% was performed until a concordance was established; defined as a difference of less than 5% between the outcomes extracted by both reviewers. Hereafter, dual data extraction was done for articles at 10% intervals, adding extra articles when in disagreement. This method resulted in a total dual data extraction rate of 29% of the included studies.

### Outcome extraction

For this study, an outcome (i.e. what was reported) was defined in alignment with the COMET initiative as ‘a measurement or observation used to capture and assess the effect of treatment such as assessment of side effects (risks) or effectiveness (benefits)’ (Williamson et al., 2017).

The primary data items extracted from each study were as follows:

- All individually named outcomes following masculinizing gGAS as stated in the PICO. These were extracted verbatim. If a study reports outcomes of multiple gender affirming surgeries, only the outcomes of the masculinizing gGAS, as stated in the PICO, were extracted;
- The number of defined individual named outcomes and the specific definitions that were used. If defined by citation, both the outcome definition and the corresponding citation were directly extracted from the cited study;
- Time of outcomes assessment, if stated;
- The instrument used to measure the outcome (OMI) (e.g. information regarding any scale, tool, classification, questionnaire or description of how an outcome was measured);
- The primary outcome used to power the study, if declared.

Furthermore secondary data items (i.e. study characteristics) were recorded:

- Title;
- Year of publication;
- Authors;
- Country where research was conducted;
- Study design; (*Oxford Center for Evidence-Based Medicine, Levels of Evidence Working Group. Study designs*, 2021)
- Number of participants undergoing masculinizing gGAS;
- Study population (i.e. transgender men and/or gender diverse individuals);
- Mean age at time of intervention;
- Intervention(s) and comparator details: type(s) of surgery (i.e. phalloplasty, metoidioplasty, scrotoplasty, coronaplasty, with- or without urethral lengthening, one- or multi staged, primary or revision/secondary procedure).

#### Outcome measurement instruments

Multiple outcomes were evaluated using questionnaires and/or multi-dimensional outcome scores that had been published previously. In these cases, each distinct question response was treated as a separate outcome. Furthermore, the overall scores from the questionnaires were deemed as additional individual outcomes. Ad-hoc or novel OMI’s, designed by researchers for a particularly study, are regularly challenging to reproduce. In those cases, each questionnaire response or score was extracted as a separate outcome (Mokkink et al., 2018).

### Synthesis of results

The taxonomy developed by Dodd et al. (2018) (Dodd et al., 2018) for the classification of outcomes included in trials is widely used in the development of COSs and endorsed by COMET (COMET Initiative, 2018). It categorizes outcomes into 38 outcome domains within five core areas to enable structured analysis. When an outcome could be categorized into multiple domains, the most appropriate and specific domain was selected. The authors of this study agreed that the outcome domain ‘25: physical functioning’ lacked granularity to cover the broad spectrum of sexual outcomes that are often reported in masculinizing gGAS research. For this systematic review, we adapted the taxonomy by adding domain ‘25B: sexual function’.

After alphabetizing all outcomes within a domain, all outcomes with exact or similar spelling were aggregated and verbatim outcomes were condensed into outcomes with similar meaning. Subsequent, the aggregated and condensed outcomes were then grouped into unique outcomes using an iterative approach. The unique outcomes were then named using the most common and/or representative wording.

### Risk of bias assessment

Evaluating the risk of bias involved the examination of both inter- and intra-study biases. This was done by extracting and analyzing the (if declared) primary outcomes. Predefining primary outcomes signifies authors’ attempts to avoid outcome reporting bias (e.g. when authors selectively publish a subset of outcomes, typically because they show significance) (Dwan et al., 2010).

## Results

### Study selection

**Figure 1.**
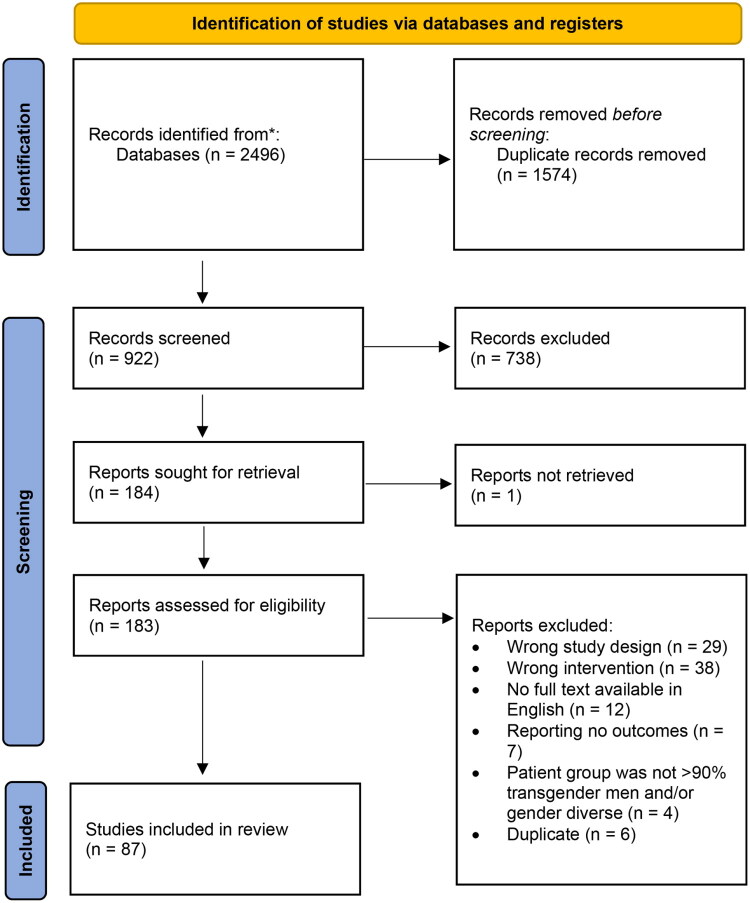
Study selection as per the PRISMA statement flow diagram (Page et al., 2021).

After removal of duplicates, a total of 922 studies were screened based on title and abstract, after which the full text was reviewed of 183 remaining articles. Finally, 87 studies were included for data extraction (Figure 1). The inter-rater consistency prior to cross-checking was high, with a Cohen’s Kappa statistic of 0.82.

### Study characteristics

An overview of the study characteristics (*n* = 87) is given in Table 1. The primary outcome was stated in 36 (41%) of the included studies.

**Table 1.** Study characteristics.

			Number of studies (%)
**Year of publication**	2020-present		33 (38)
	2015–2020		14 (16)
	2010–2015		8 (9)
	2005–2010		21 (24)
	2000–2005		2 (2)
	<2000		9 (10)
		**References**	
**Country where study was conducted**	*United States of America*	(Ascha et al., 2018; Cylinder et al., 2022; Danker et al., 2020; Ha et al., 2022; Lin-Brande et al., 2021; Meltzer & Esmonde, 2020; Miller et al., 2021; Noe et al., 1974; Odeluga et al., 2021; Peters et al., 2022; Robinson et al., 2021; 2023; Salgado et al., 2021; Santucci et al., 2021; Saxena et al., 2023; Sengezer & Sadove, 1993; Yuan et al., 2022)	17 (20)
	*The Netherlands*	(Al-Tamimi, Pigot, Ronkes, et al., 2020; Al-Tamimi et al., 2019; de Rooij et al., 2021; 2023; Elfering et al., 2019; 2021; Hage, 1996; Hage et al., 1993; Hage & van Turnhout, 2006; Pigot et al., 2020; van de Grift et al., 2017; 2019; van der Sluis et al., 2017; Veerman et al., 2020)	14 (16)
	*Germany*	(Küenzlen et al., 2020; 2022; Papadopulos et al., 2001, 2008; 2021; Schaff, 2007; Schaff & Papadopulos, 2009; Spennato et al., 2022; Wirthmann et al., 2018)	10 (11)
	*Belgium*	(Doornaert et al., 2011; Monstrey et al., 2005; 2009; Selvaggi et al., 2006; 2009; Van Caenegem et al., 2013; Waterschoot et al., 2021; Wierckx et al., 2011)	8 (9)
	*Serbia*	(Bordas et al., 2021; Djordjevic, Bencic, et al., 2019; Djordjevic, Bizic, et al., 2009; Djordjevic, Stanojevic, et al., 2009; Djordjevic & Bizic, 2013; Perovic & Djordjevic, 2003; Stojanovic et al., 2017; Vukadinovic et al., 2014)	8 (9)
	*United Kingdom*	(Dabernig et al., 2006; Garaffa, Christopher, & Ralph, 2010; Garaffa, Ralph, & Christopher, 2010; Garcia et al., 2014; Matti et al., 1988)	5 (6)
	*Other*	(Akhoondinasab et al., 2020; Bettocchi et al., 2005; Cheng et al., 2021; Cohanzad, 2016; Falcone et al., 2020; Fang et al., 1994; 1998, 1999; Felici & Felici, 2006; Gao et al., 2022; Gupta et al., 2022; Kim et al., 2009; 2010; Kjölhede et al., 2019; Legaillard et al., 1995; Leriche et al., 2008; Namba et al., 2019; Ranno et al., 2007; Song et al., 2011; Staud et al., 2021; Takamatsu & Harashina, 2009; Terrier et al., 2014; 2020; Vesely et al., 2007; Zhang et al., 2015)	25 (29)
**Study design**	Cohort		72 (83)
	Case series		7 (8)
	Case-control		1 (1)
	Cross-sectional		4 (5)
	RCT		1 (1)
	Longitudinal		1 (1)
	Mixed-method		1 (1)
**Study population**	Transgender men only		86 (99)
	Transgender men and gender diverse individuals		1 (1)
	Gender diverse individuals only		0 (0)
**Sample size**			
Number of participants undergoing masculinizing gGAS	Mean (SD)		76 (113)
	Median (range)Total		40 (2–815)6610
**Reported mean age at time of intervention**	Median (range)		33 (24–45)
	Not stated		17 studies

### Surgical interventions

The included studies reported a total number of 13,433 surgical interventions: 4973 scrotoplasties, 4548 phalloplasties (of which 3834 with urethral lengthening), 1895 metoidioplasties (of which 1731 with urethral lengthening) and 2017 coronaplasties. The types of skin flaps used for the phalloplasty and metoidioplasty techniques (with- and without urethral lengthening) are presented in Table 2. Eighty-two studies reported on primary interventions, one on a secondary intervention and 4 on both. Fifty (57%) studies reported on one stage surgery, 36 (41%) on staged and 1 study did not clearly state whether the surgical intervention was staged or not.

**Table 2.** Phalloplasty and metoidioplasty (with- and without urethral lengthening) technique specifications.

Phalloplasty technique	Frequency reported	Phalloplasty urethral lengthening technique	Frequency reported	Metaidoioplasty technique	Frequency reported
FRFF	2937	FRFF twt	2126	Metaidoioplasty (not further specified)	1640
pALT	496	FRFF twt + vaginal flap + labia minora flap	320	Metaidoioplasty Hage’s	128
Abdominal flap	257	FRFF twt + labia minora flap	154	Metaidoioplasty Labial ring	117
SCIP	241	FRFF	101	Metaidoioplasty extensive	10
LD	181	Labia minora flap + vaginal flap	100	**Total**	**1895**
Fibula flap	129	Buccal mucosa	92		
fALT	25	Vaginal flap	91	**Metaidoioplasty urethral lengthening technique**	**Frequency reported**
SIEA	15	Labia minora flap	80	Labia minora flap	651
FRFF + DIEP	6	Labia minora flap + clitoral skin flap	80	Buccal mucosa + genital skin grafts	400
Gracilis muscle flap	5	FRFF twt + vaginal flap + labia minora flap + local skin flap	72	Buccal mucosa + labial minora flap	201
Rectus abdominis myocutaneous pedicled	4	pALT twt + labia minora flap	64	Vaginal mucosa + vestibular skin flaps + labial flap	102
FRFF + pALT	3	Split skin graft	56	Metaidoioplasty	100
DIEP	2	Vaginal mucosa	52	Vaginal flap + labial minora flap	70
DIEP + pALT	2	Labia minora flap + clitoral skin flap + vaginal flap	47	Vaginal flap	59
Groin flap	2	SCIP	43	Buccal mucosa + clitoral skin flap	49
Lateral upper arm flap	1	pALT twt + vaginal flap + labia minora flap	41	Onlay flap + genital skin graft	42
Not stated	242	pALT twt + vaginal flap + labia minora flap + local skin flap	41	Transverse preputial skin island	34
		pALT twt + pedicled mucosa flaps	39	Buccal mucosa	20
		Full thickness skin graft	26	Not stated	3
		fALT	19	**Total**	**1731**
		pALT twt	12		
		Vaginal mucosa + local skin flaps	10		
		fALT twt	9		
		Fibula flap twt	8		
		pALT	6		
		Clitoral skin flap	2		
		Metaidoioplasty	2		
		Not stated	141		
**Total**	**4548**	**Total**	**3834**		

DIEP = Deep Inferior Epigastric Perforator, pALT = pedicled Anterior Lateral Thigh, fALT = free Anterior Lateral Thigh, FRFF = Free Radial Forearm Flap, LD = Latissimus Dorsi, SCIP = Superficial Circumflex Iliac Perforator, SIEA = Superficial Inferior Epigastric Artery, twt = tube-within-tube.

#### Outcomes

**Figure 2.**
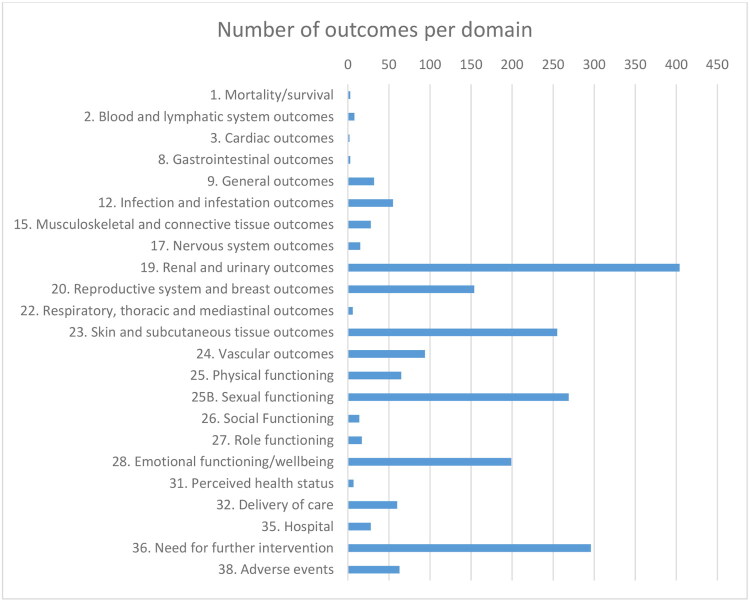
Number of individual outcomes per domain.

A total number of 2077 individual outcomes were extracted, of which 1026 (49%) were defined. The reported outcomes could be categorized into 22 domains (Figure 2). Time of outcome assessment was mentioned for 1116 (54%) outcomes. However, time of assessment was often described by an indication of the post-operative clinical follow-up period, which varied from periods of months to several years (e.g. ‘5–53 months post-operative’ or ‘average 68,52 months post-operative’). Similarly, studies used terms like ‘early’ and ‘late’ complication, without defining these time periods. Specific time of assessment descriptions, such as ‘within 24 h post-operative’, ‘0–30 days post-operative’ and ‘at one year post-operative’, were reported for 486 (23%) outcomes.

The process of aggregating and condensing the initial 2077 individual outcomes generated a total of 384 unique outcomes. Table 3 below presents the top 10 most frequently reported unique outcomes, along with their reporting frequency and the number of times they were defined. Additionally, for each unique outcome, several examples are shown of individual outcome descriptions and provided definitions. The latter illustrates significant variability in the incorporation of different time frames, measurement instruments used, and outcome management within the outcome definitions.

**Table 3.** Top 10 most reported outcomes.

Unique outcome	Reporting frequency (%)	Of which defined (%)	Examples of individual outcome descriptions	Examples of provided outcome definitions
Neo-urethral fistula	104 (27)	32 (31)	Fistula formation, Fistulisation, Urethrocutaneous fistula, Neo-urethral fistula, Urinary fistula, Fistulae	“Any abnormal connection between skin and urethra (including meatal dehiscence) that was not closed spontaneously within 3 months after surgery or that occurred at a later stage” (Waterschoot et al., 2021), “A urethrocutaneous connection that persisted for or occurred more than 6 wk after surgery and required catheterization and/or surgical treatment” (Veerman et al., 2020), “Urethrocutaneous fistula developed immediately after spontaneous micturition through neo-urethra” (Takamatsu & Harashina, 2009)
Neo-urethral stricture	81 (21)	26 (32)	Stenosis, Neo-urethral stricture, Urethral contracture, Urethrostenosis, Urologic stricture, Stricture	“Urethral stricture was defined as narrowing of the urethra at retrograde urethrogram during a period of obstructive voiding (weak urinary stream, straining and Q-max less than 15 ml per second)” (Veerman et al., 2020), “Any narrowing at the urethra that impeded passage of a 16 F cystoscope” (Waterschoot et al., 2021), “Urethral stricture classified as major complication, which could be managed by a minor surgical procedure” (Djordjevic, Bizic, et al., 2009; Djordjevic, Stanojevic, et al., 2009; Djordjevic & Bizic, 2013)
Partial flap necrosis	56 (15)	21 (38)	Areas of skin necrosis, Flap distal limited necrosis, Partial flap loss, Partial flap necrosis, Tip neo-phallus necrosis	“Partial flap necrosis was defined as any patient requiring sharp excision of necrotic flap tissue. Superficial epidermolysis was not included” (Cylinder et al., 2022), “Full-thickness skin necrosis making up <10% of the total flap size” (Monstrey et al., 2009), “Limited to the distal part of the neophallus, healed spontaneously with minimal lost in length” (Djordjevic, Bencic, et al., 2019), “At the top of the phallus, needing debridement and coverage with a split skin graft” (Pigot et al., 2020)
Complete flap necrosis	50 (13)	3 (6)	Complete flap failure, Total flap necrosis, Loss of phalloplasty	“Complete flap loss for which salvage phalloplasty was performed at a later time” (Pigot et al., 2020), “Total flap necrosis due to vein thrombosis” (Djordjevic, Bencic, et al., 2019)
Tactile sensibility in the neo-phallus	36 (9)	24 (67)	Cutaneous sensitivity in phalloplasty, Phallic tactile sensibility, Presence of touch sensation in neophallus	“Penile shaft pressure perception, sharp-blunt discrimination and two-point discrimination” (Kuenzlen et al., 2020), “Experience of tactile sensation in neo-phallus (defined by citation (Bubanj et al., 2004))” (Djordjevic, Bencic, et al., 2019), “Frequency of experiencing tactile sensitivity in the neo-phallus in the last 4 wk” (Elfering et al., 2021)
Hematoma	36 (9)	4 (11)	Flap hematoma, Heamatoma, Hematoma	“Hematoma (classified as Clavien Dindo grade I-II (Clavien et al., 2009))” (Terrier et al., 2020), “Hematoma requiring surgical drainage” (Pigot et al., 2020)
Need for re-intervention neo-urethral fistula	34 (9)	1 (3)	Revision for fistula, Re-operation for urethral fistula, Fistulectomy, Minor surgery for fistula	“Closing fistula under local anesthesia as an outpatient procedure” (Takamatsu & Harashina, 2009)
Need for re-intervention neo-urethral stricture	34 (9)	0 (0)	Urethral stricture needing revision, Stricture needing surgical repair, Minor revision for stricture	N/A.
Ability to perform penetrative sexual intercourse	33 (9)	19 (58)	Ability to have penetrative sexual intercourse, Ability to penetrate during sexual intercourse, Use of penis for penetration	“Sexual intercourse with penetration without erectile prosthesis” (Djordjevic, Bencic, et al., 2019), “Length neo-phallus adequate for full penetration” (Vukadinovic et al., 2014), “Ability to use minipenis for intercourse with partner” (Takamatsu & Harashina, 2009)
Ability to void in a standing position	33 (9)	7 (21)	Ability to stand and pass urine, Stand to pee, Ability to void while standing	“Ability to void standing up through the opened fly” (Hage, 1996), “The ability to void from the tip of the phallus while standing, with no irritative or obstructive urinary symptoms, and with minimal residual bladder urine of 30 mL, as assessed using ultrasonography” (Garaffa, Ralph, & Christopher, 2010)

An overview of the unique outcomes reported 10 times or more is provided in supplementary material 2. These unique outcomes (covering 1149 individual outcomes) constitute 55% of all reported outcomes. Complications and re-interventions for complications account for 648 of these outcomes, representing over half of the outcomes reported 10 times or more. These fall mostly in the domains urinary functioning, skin and subcutaneous tissue and need for re-intervention. Approximately one-third (34%) are patient-reported outcomes, with half of these falling under the domain 25B: Sexual functioning. Within the unique outcomes reported 10 times or more, the difference in the degree of outcome specificity is demonstrated among the outcomes related to orgasming: specifically, ‘ability to orgasm’, ‘ability to achieve orgasm by masturbation’, and ‘ability to achieve orgasm during sexual intercourse with a partner’.

#### Outcome measurement instruments

The description of the used OMI was provided for 987 (48%) outcomes, while 559 (57%) of the OMIs were novel or ad hoc, developed for the purpose of that study. In Table 4, the OMIs that were reported at least 10 times are presented. Among the novel/ad hoc OMIs employed, Likert scales emerged as the most commonly used across studies, frequently measuring patient-reported outcomes. These scales were applied in various manners, ranging from assessing the frequency of an outcome (e.g. never—sometimes—regularly—often—always) to measuring the level of satisfaction with an outcome (e.g. completely dissatisfied—somewhat dissatisfied—neutral—somewhat satisfied—completely satisfied). The presence or absence of specific outcomes could also be reported as a binary rating (e.g. yes/no). Out of the 987 outcomes for which an OMI was provided, 43% were assessed using OMIs that had been previously published, commonly used, or validated. In this category the highest number of outcomes were measured using the ‘Fragen zur Lebens Zufriedenheit’ (FLZ) Questions on Life Satisfaction Modules (Henrich & Herschbach, 2000), although it was only used in two studies. On the other hand, the Clavien-Dindo classification (Clavien et al., 2009), used to categorize complications or adverse events, was used in five studies, but for fewer outcomes. A modified Mackinnon Pain Questionnaire (Bailey et al., 2009; Chen et al., 1998; Domeshek et al., 2017; Heary et al., 2022; Wojtkiewicz et al., 2015) was used to assess donor-site pain in one study. To assess donor-site scar-related outcomes, researchers have used the Patient and Observer Scar Assessment Scale (POSAS) (Carrière et al., 2023) and the Vancouver Scar Scale (VSS) (Baryza & Baryza, 1995). The International Prostate Symptom Score (IPSS) (Barry et al., 2017), uroflowmetry, retrograde urethrocystography, cystoscopy, and frequency voiding chart were used to measure the majority of the reported urological outcomes of included studies. Furthermore, uroflowmetry and retrograde urethrocystography were the most used OMIs for clinical outcomes across studies.

**Table 4.** Outcome measurement instruments.

	Frequency used to measure individual outcomes (%)	Frequency used by studies (%)
Outcome measurement instrument		
FLZ Questions on Life Satisfaction Modules^24^	54 (13)	2 (2)
Clavien-Dindo classification^25^	49 (11)	5 (6)
Modified Mackinnon Pain Questionnaire (Bailey et al., 2009; Chen et al., 1998; Domeshek et al., 2017; Heary et al., 2022; Wojtkiewicz et al., 2015)	36 (8)	1 (1)
Uroflowmetry (not further specified)	35 (8)	8 (9)
International Prostate Symptom Score^28^	20 (5)	4 (5)
Histological- staining and examination using a light microscope	18 (4)	1 (1)
OSAS of the POSAS^26^	14 (3)	2 (2)
PSAS of the POSAS^26^	14 (3)	2 (2)
Retrograde urethrocystography	13 (3)	8 (9)
Goniometer (not further specified)	11 (3)	1 (1)
Frequency voiding chart	10 (2)	3 (3)
Cystoscope 13 F	10 (2)	6 (7)
**Novel/Ad hoc Outcome Measurement Instrument**		
Patient rating outcome on 5-point Likert scale	217 (39)	11 (13)
Patient rating outcome on 3-point Likert scale	112 (20)	10 (11)
Patient rating outcome binary (yes/no)	94 (17)	8 (9)
Patient interview using open questions	43 (8)	5 (6)
Patient rating outcome on 4-point Likert scale	28 (5)	6 (7)
Clinician and patient outcome rating on numeric scale 1–10	13 (2)	2 (2)
Novel patient questionnaire (not further specified)	13 (2)	2 (2)

## Discussion

This systematic review aimed to provide a comprehensive overview of all outcomes that have been reported in clinical studies reporting on masculinizing gGAS procedures in TGD individuals. The results confirmed significant heterogeneity in the reported outcomes, their definitions, degree of specificity, the time of assessment and the measurement instruments used. Moreover, studies omitted to report definitions, timing of assessment and the used OMI for approximately half of the in total 2077 reported outcomes. Only one-third of the studies declared a primary outcome, indicating a high risk of bias. Overall, the results suggest a low level of evidence among the included studies.

### Surgical interventions

Together, the included studies reported on a total of 6,610 included TGD individuals and 13,433 performed surgical interventions. However, these numbers are likely an overestimation, as some studies by the same author reported overlapping inclusion periods, potentially reporting on the same intervention and TGD individual more than once.

A large variety of surgical techniques were employed, including the use of different skin flaps for reconstructions and variations in the staging of operations. However, descriptions of the surgical techniques are often ambiguous and/or incomplete. As a result, the degree of similarity between the techniques used in different studies is unclear. Most of the included studies were retrospective cohort studies and focused on assessing a specific surgical technique. Nonetheless, authors rarely described specific indications for treatment, TGD individual selection criteria, or a rationale for using the chosen surgical technique. Therefore, these studies provide limited insight into which treatment is appropriate for individual TGD persons.

#### Outcomes, time of outcomes assessment and measurement instrument

The vast majority of outcomes were clinical outcomes, which comprised mainly adverse events and functional outcomes. Adverse events were mostly related to urologic and flap complications and their management. Functional outcomes related mainly to urological and sexual function. Accordingly, the majority of reported outcomes were in the domains of renal and urinary function, re-interventions and (sub)cutaneous tissue. This is not surprising, since the occurrence rate of adverse events in these domains is high in masculinizing gGAS (Cylinder et al., 2022; Hu et al., 2022; Wang et al., 2022). The consistent reporting pattern of adverse events should be recognized as a valuable effort, as these outcomes are critical for informed consent and surgical treatment education. However, it remains unclear whether this pattern emerged primarily to attempt transparency about complications or if it also reflects the researchers’ interests or surgeons’ views on which outcomes most effectively evaluate the surgery. This uncertainty is further supported by the majority of included studies being of retrospective design and only a minority declaring a primary outcome. Furthermore, few studies research how these complications are experienced by individuals post-surgery, or do so in the context of ‘regret’ or ‘recommending the surgery to others’. Patient-Reported Outcomes (PROs) accounted for about a third of the reported unique outcomes, mainly within the sexual function domain. Most PROs assessed the *ability* to perform certain sexual functions, but few assessed how these functional outcomes are *experienced*. Furthermore, it is noteworthy that cis-normative functional outcomes, such as the ability to void in a standing position and the ability to perform penetrative sexual intercourse, are among the most frequently reported PROs, despite being achievable for only a part of those receiving masculinizing gGAS.

The lack of PROs and outcomes from a TGD individuals perspective results in a lack of knowledge about treatment efficacy and further hinders the personalized selection of the best masculinizing gGAS technique. The extent of the heterogeneity becomes clear when looking more closely at the way in which the outcomes are reported. Firstly, the degree of outcome specificity varied, as previously illustrated with the outcomes related to orgasming. Secondly, for only half of the outcomes definitions were provided. However, these definitions were inconsistent and the variability is evident. Differences in incorporated time frames can significantly influence reported incidence rates. For instance, definitions of neo-urethral fistula range from occurring ‘after six weeks’ to ‘within three months’. Additionally, grading within definitions, like the use of the Clavien-Dindo classification, such as in hematomas, introduces varying degrees of severity that may not be consistently reported across studies. Furthermore, definitions of partial flap necrosis not only vary in the extent of tissue loss but also in the management strategies employed, which may impact recovery and long-term outcomes. Moreover, the inclusion of both clinical criteria and patient-reported experiences in defining outcomes, as seen with neo-urethral stricture, tactile sensibility in the neo-phallus and ability to void in a standing position, underscores the diverse approaches.

Besides the variability in definitions, there was also a large variation in how and when outcomes were measured. Most Patient Reported Outcome Measures (PROMs) used were novel/ad hoc questionnaires, constituting more than half of the reported OMIs, or questionnaires that were not validated for the transgender and gender diverse population. Additionally, clinical symptom indexes, such as the IPSS (Barry et al., 2017), were employed in several studies, despite not being validated for the population. A total of 49 outcomes were reported using the Clavien-Dindo classification of surgical complications. While widely implemented across surgical research fields (Clavien et al., 2009), it was only used for a marginal number of the reported surgical complication outcomes among the included studies. For 77% of the reported outcomes, the time of outcome assessment was not reported or was vaguely described. The timing of outcome measurements varied between studies, sometimes differing by months or even years. Furthermore, some studies measured outcomes at a single time point, while others measured them at multiple times.

Currently, there is insufficient evidence to confidently compare how surgical techniques differ in terms of safety and effectiveness of outcomes, or to determine the most appropriate surgical treatment for a TGD individual. Although the number of studies on masculinizing gGAS has been increasing rapidly over recent years, the heterogeneity of reported outcomes on all levels, the reporting bias, and the mainly retrospective study design prevents progress in this field.

The need for standardization in research on gender-affirming surgery has been widely recognized (Cylinder et al., 2022; Hu et al., 2022; Morrison et al., 2016; Wang et al., 2022), and this systematic review highlights this urgent need in masculinizing gGAS research. This review is a starting point for The GenderCOS project, which aims to develop a COS as the next step toward more evidence-based gender-affirmative surgical care. This will be done by reaching international, multi-stakeholder consensus on a set of well-defined outcomes that should, as a minimum, be consistently measured and reported in all clinical trials relating to masculinizing gGAS (Roijer et al., 2023).

#### Strengths and limitations

This systematic review represents a complete overview of outcome measurement in masculinizing gGAS studies to date. The search strategy was broad to maximize inclusion of relevant studies and the current study was comprehensive in its data gathering. All outcomes, definitions, times of outcome assessment and measurement instruments used, were gathered and presented for a wide masculinizing gGAS scope and thus leaving little chance for missing any relevant results needed for the development of a COS. However, aggregation of outcomes contains a subjective component giving the high variability of outcomes and heterogeneity of definitions used.

This systematic review is limited by the level of evidence of the included studies. With (retrospective) cohort studies and case series being the most commonly used study designs among the included studies, there is a notable susceptibility to reporting bias. Although the search strategy was not restricted by language, resources were not available to include full text non-English studies, therefor unable to minimize the risk of language bias.

Our results are similar to those of an analogous review regarding feminizing genital gender-affirming surgery (Pidgeon et al., 2023). However, the situation for masculinizing gGAS is even worse, with even less complete information regarding the outcomes used. This reiterates the need to develop widely supported COS for both feminizing gGAS and masculinizing gGAS (Roijer et al., 2023).

## Conclusions

This systematic review identified significant heterogeneity in studies reporting on masculinizing gGAS, with great variety in reported outcomes, their definitions, degrees of specificity, the time of assessment, and the measurement instruments used. Additionally, reporting was frequently incomplete and susceptible to bias, primarily due to the retrospective study designs. The adoption of a standardized set of outcomes and definitions (a Core Outcome Set) in future masculinizing gGAS research is essential. This will enable treatment comparisons and improve evidence-based practice and decision-making. Ultimately, this will improve personalized surgical care for the transgender and gender diverse population.

## Supplementary Material

Supplemental Material
