# Supplementary material for: Navigating toward standardization: A systematic review mapping outcome measures in masculinizing genital gender affirming surgery
Source: Int J Transgend Health. 2024 Nov 20;27(2):1086–103. doi: 10.1080/26895269.2024.2429490 (PMC13015112; doi:10.1080/26895269.2024.2429490)
Supplement: Supplemental Material [file WIJT_A_2429490_SM4004.docx]

Supplementary material 1: search strategy

**Search strategy for Ovid/Medline (21 September 2023)**

| **1** | (metoidioplast* or metaidioplast* or phalloplast* or scrotoplast* or neo-phall* or neophall* or coronaplast* or corona-plast* or glans-plast* or glansplast* or (masculin* adj3 (genitoplast* or genital*))).ti,ab,kf. | **1,079** |
| --- | --- | --- |
| **2** | ((penile or penis) adj3 reconstruct*).ti,ab,kf. | **760** |
| **3** | exp Sex Reassignment Procedures/ or exp Gender Dysphoria/ or exp Transgender Persons/ or exp Transsexualism/ or (gender-disorder* or gender-identity-disorder* or gender-dysphor* or sexual-dysphor* or sex-dysphor* or gender-incongruen* or transgender* or trans-gender* or transsex* or trans-sex* or sex-reassign* or sex-change* or gender-reassign* or gender-chang* or gender-affirm* or transwom* or trans-wom* or transman or transmen or trans-man or trans-men or transmale* or trans-male* or female-to-male or assigned-female-at-birth or assigned-female-sex or biological female* or biological-women or transboy* or trans-boy* or gender-divers*).ti,ab,kf. | **23,427** |
| **4** | (1 or 2) and 3 | **534** |

##

**Search strategy for Embase.com (21 September 2023)**

| **No.** | **Query** | **Results** |
| --- | --- | --- |
| **#5** | #3 NOT #4 | **555** |
| **#4** | #3 AND ('conference abstract'/it OR 'conference paper'/it OR 'conference review'/it) | **254** |
| **#3** | #1 AND #2 | **809** |
| **#2** | 'sex reassignment'/exp OR 'gender dysphoria'/exp OR 'transgender'/de OR 'female to male transgender'/exp OR 'gender disorder*':ti,ab,kw OR 'gender identity disorder*':ti,ab,kw OR 'gender dysphor*':ti,ab,kw OR 'sexual dysphor*':ti,ab,kw OR 'sex dysphor*':ti,ab,kw OR 'gender incongruen*':ti,ab,kw OR transgender*:ti,ab,kw OR 'trans gender*':ti,ab,kw OR transsex*:ti,ab,kw OR 'trans sex*':ti,ab,kw OR 'sex reassign*':ti,ab,kw OR 'sex change*':ti,ab,kw OR 'gender reassign*':ti,ab,kw OR 'gender chang*':ti,ab,kw OR 'gender affirm*':ti,ab,kw OR transwom*:ti,ab,kw OR 'trans wom*':ti,ab,kw OR transman:ti,ab,kw OR transmen:ti,ab,kw OR 'trans man':ti,ab,kw OR 'trans men':ti,ab,kw OR transmale*:ti,ab,kw OR 'trans male*':ti,ab,kw OR 'female to male':ti,ab,kw OR 'assigned female at birth':ti,ab,kw OR 'assigned female sex':ti,ab,kw OR 'biological female*':ti,ab,kw OR 'biological women':ti,ab,kw OR transboy*:ti,ab,kw OR 'trans boy*':ti,ab,kw OR 'gender divers*':ti,ab,kw | **32,854** |
| **#1** | 'metoidioplasty'/exp OR 'scrotoplasty'/exp OR 'phalloplasty'/exp OR 'neophallus'/exp OR 'glansplasty'/exp OR 'penis reconstruction'/exp OR metoidioplast*:ti,ab,kw OR metaidioplast*:ti,ab,kw OR phalloplast*:ti,ab,kw OR scrotoplast*:ti,ab,kw OR 'neo phall*':ti,ab,kw OR neophall*:ti,ab,kw OR coronaplast*:ti,ab,kw OR 'corona plast*':ti,ab,kw OR 'glans plast*':ti,ab,kw OR glansplast*:ti,ab,kw OR ((masculin* NEAR/3 (genitoplast* OR genital*)):ti,ab,kw) OR (((penile OR penis) NEAR/3 reconstruct*):ti,ab,kw) | **2,574** |

**Search strategy for Clarivate Analytics/Web of Science Core Collection (21 September 2023)**

| **#3** | #1 AND #2 | **631** |
| --- | --- | --- |
| **#2** | TS=("gender-disorder*" OR "gender-identity-disorder*" OR "gender-dysphor*" OR "sexual-dysphor*" OR "sex-dysphor*" OR "gender-incongruen*" OR "transgender*" OR "trans-gender*" OR "transsex*" OR "trans-sex*" OR "sex-reassign*" OR "sex-change*" OR "gender-reassign*" OR "gender-chang*" OR "gender-affirm*" OR "transwom*" OR "trans-wom*" OR "transman" OR "transmen" OR "trans-man" OR "trans-men" OR "transmale*" OR "trans-male*" OR "female-to-male" OR "assigned-female-at-birth" OR "assigned-female-sex" OR "biological-female*" OR "biological-women" OR "transboy*" OR "trans-boy*" OR "gender-divers*") | **39,516** |
| **#1** | TS=("metoidioplast*" OR "metaidioplast*" OR "phalloplast*" OR "scrotoplast*" OR "neo-phall*" OR "neophall*" OR "coronaplast*" OR "corona-plast*" OR "glans-plast*" OR "glansplast*" OR ("masculin*" NEAR/3 ("genitoplast*" OR "genital*")) OR (("penile" OR "penis") NEAR/3 "reconstruct*")) | **2,061** |

**Search strategy for Elsevier/Scopus (21 September 2023)**

| **History Count** | **Search Terms** | **Results** |
| --- | --- | --- |
| **#3** | **#1 AND #2** | **697** |
| **#2** | **TITLE-ABS-KEY (("gender-disorder*" OR "gender-identity-disorder*" OR "gender-dysphor*" OR "sexual-dysphor*" OR "sex-dysphor*" OR "gender-incongruen*" OR "transgender*" OR "trans-gender*" OR "transsex*" OR "trans-sex*" OR "sex-reassign*" OR "sex-change*" OR "gender-reassign*" OR "gender-chang*" OR "gender-affirm*" OR "transwom*" OR "trans-wom*" OR "transman" OR "transmen" OR "trans-man" OR "trans-men" OR "transmale*" OR "trans-male*" OR "female-to-male" OR "assigned-female-at-birth" OR "assigned-female-sex" OR "biological-female*" OR "biological-women" OR "transboy*" OR "trans-boy*" OR "gender-divers*"))** | **45,887** |
| **#1** | **TITLE-ABS-KEY (("metoidioplast*" OR "metaidioplast*" OR "phalloplast*" OR "scrotoplast*" OR "neo-phall*" OR "neophall*" OR "coronaplast*" OR "corona-plast*" OR "glans-plast*" OR "glansplast*" OR ("masculin*" W/3 ("genitoplast*" OR "genital*")) OR (("penile" OR "penis") W/3 "reconstruct*")))** | **2,549** |

**Search strategy for Ebsco/APA PsycINFO (21 September 2023)**

| **#** | **Query** | **Results** |
| --- | --- | --- |
| **S3** | **S1 AND S2** | **77** |
| **S2** | **DE "Transgender" OR DE "Gender Reassignment" OR DE "Gender Identity" OR DE "Gender Nonbinary" OR DE "Gender Nonconforming" OR DE "Transsexualism" OR DE "Gender Dysphoria" OR TI("gender-disorder*" OR "gender-identity-disorder*" OR "gender-dysphor*" OR "sexual-dysphor*" OR "sex-dysphor*" OR "gender-incongruen*" OR "transgender*" OR "trans-gender*" OR "transsex*" OR "trans-sex*" OR "sex-reassign*" OR "sex-change*" OR "gender-reassign*" OR "gender-chang*" OR "gender-affirm*" OR "transwom*" OR "trans-wom*" OR "transman" OR "transmen" OR "trans-man" OR "trans-men" OR "transmale*" OR "trans-male*" OR "female-to-male" OR "assigned-female-at-birth" OR "assigned-female-sex" OR "biological-female*" OR "biological-women" OR "transboy*" OR "trans-boy*" OR "gender-divers*") OR AB("gender-disorder*" OR "gender-identity-disorder*" OR "gender-dysphor*" OR "sexual-dysphor*" OR "sex-dysphor*" OR "gender-incongruen*" OR "transgender*" OR "trans-gender*" OR "transsex*" OR "trans-sex*" OR "sex-reassign*" OR "sex-change*" OR "gender-reassign*" OR "gender-chang*" OR "gender-affirm*" OR "transwom*" OR "trans-wom*" OR "transman" OR "transmen" OR "trans-man" OR "trans-men" OR "transmale*" OR "trans-male*" OR "female-to-male" OR "assigned-female-at-birth" OR "assigned-female-sex" OR "biological-female*" OR "biological-women" OR "transboy*" OR "trans-boy*" OR "gender-divers*") OR KW("gender-disorder*" OR "gender-identity-disorder*" OR "gender-dysphor*" OR "sexual-dysphor*" OR "sex-dysphor*" OR "gender-incongruen*" OR "transgender*" OR "trans-gender*" OR "transsex*" OR "trans-sex*" OR "sex-reassign*" OR "sex-change*" OR "gender-reassign*" OR "gender-chang*" OR "gender-affirm*" OR "transwom*" OR "trans-wom*" OR "transman" OR "transmen" OR "trans-man" OR "trans-men" OR "transmale*" OR "trans-male*" OR "female-to-male" OR "assigned-female-at-birth" OR "assigned-female-sex" OR "biological-female*" OR "biological-women" OR "transboy*" OR "trans-boy*" OR "gender-divers*")** | **44,774** |
| **S1** | **TI("metoidioplast*" OR "metaidioplast*" OR "phalloplast*" OR "scrotoplast*" OR "neo-phall*" OR "neophall*" OR "coronaplast*" OR "corona-plast*" OR "glans-plast*" OR "glansplast*" OR ("masculin*" N3 ("genitoplast*" OR "genital*")) OR (("penile" OR "penis") N3 "reconstruct*")) OR AB("metoidioplast*" OR "metaidioplast*" OR "phalloplast*" OR "scrotoplast*" OR "neo-phall*" OR "neophall*" OR "coronaplast*" OR "corona-plast*" OR "glans-plast*" OR "glansplast*" OR ("masculin*" N3 ("genitoplast*" OR "genital*")) OR (("penile" OR "penis") N3 "reconstruct*")) OR KW("metoidioplast*" OR "metaidioplast*" OR "phalloplast*" OR "scrotoplast*" OR "neo-phall*" OR "neophall*" OR "coronaplast*" OR "corona-plast*" OR "glans-plast*" OR "glansplast*" OR ("masculin*" N3 ("genitoplast*" OR "genital*")) OR (("penile" OR "penis") N3 "reconstruct*"))** | **132** |

**Search strategy for ProQuest/International Bibliography of Social Sciences (IBSS) (21 September 2023)**

| **#** | **Query** | **Results** |
| --- | --- | --- |
| **#3** | **#1 AND #2** | **2** |
| **#2** | **NOFT("gender-disorder*" OR "gender-identity-disorder*" OR "gender-dysphor*" OR "sexual-dysphor*" OR "sex-dysphor*" OR "gender-incongruen*" OR "transgender*" OR "trans-gender*" OR "transsex*" OR "trans-sex*" OR "sex-reassign*" OR "sex-change*" OR "gender-reassign*" OR "gender-chang*" OR "gender-affirm*" OR "transwom*" OR "trans-wom*" OR "transman" OR "transmen" OR "trans-man" OR "trans-men" OR "transmale*" OR "trans-male*" OR "female-to-male" OR "assigned-female-at-birth" OR "assigned-female-sex" OR "biological-female*" OR "biological-women" OR "transboy*" OR "trans-boy*" OR "gender-divers*")** | **6,562** |
| **#1** | **NOFT("metoidioplast*" OR "metaidioplast*" OR "phalloplast*" OR "scrotoplast*" OR "neo-phall*" OR "neophall*" OR "coronaplast*" OR "corona-plast*" OR "glans-plast*" OR "glansplast*" OR ("masculin*" NEAR/3 ("genitoplast*" OR "genital*")) OR (("penile" OR "penis") NEAR/3 "reconstruct*")** | **8** |

Supplementary material 2: table containing unique outcomes reported 10 times or more.

| **Unique outcomes** | **Number of reporting** | **Of which clearly defined** | **Outcome domain** | **Complication, re-intervention for complication, PRO or other** |
| --- | --- | --- | --- | --- |
| Urethral fistula | 104 | 32 | 19. Renal and urinary outcomes | Complication |
| Urethral stricture | 81 | 26 | 19. Renal and urinary outcomes | Complication |
| Partial flap necrosis | 56 | 21 | 23. Skin and subcutaneous tissue outcomes | Complication |
| Complete flap necrosis | 50 | 3 | 23. Skin and subcutaneous tissue outcomes | Complication |
| Tactile sensibility neo-phallus | 36 | 24 | 20. Reproductive system and breast outcomes | PRO |
| Hematoma | 36 | 4 | 24. Vascular outcomes | Complication |
| Need for re-intervention urologic fistula | 34 | 1 | 36. Need for further intervention | Re-intervention for complication |
| Need for re-intervention urologic stricture | 34 | 0 | 36. Need for further intervention | Re-intervention for complication |
| Ability to perform penetrative sexual intercourse | 33 | 19 | 25B. Sexual functioning | PRO |
| Ability to void in standing position | 33 | 7 | 19. Renal and urinary outcomes | PRO |
| Need for re-reintervention (not further specified) | 33 | 2 | 36. Need for further intervention | Other |
| Satisfaction with neo-genital esthetic result | 32 | 24 | 28. Emotional functioning/wellbeing | PRO |
| Length of hospital stay | 27 | 1 | 35. Hospital | Other |
| Donor-site pain | 27 | 26 | 9. General outcomes | Complication |
| Satisfaction with voiding ability | 23 | 14 | 19. Renal and urinary outcomes | PRO |
| Ability to achieve orgasm by masturbation | 22 | 21 | 25B. Sexual functioning | PRO |
| Length neo-phallus | 21 | 5 | 20. Reproductive system and breast outcomes | Other |
| Need for re-intervention vascular complication | 21 | 1 | 36. Need for further intervention | Re-intervention for complication |
| Satisfaction with neo-phallus esthetic result | 21 | 12 | 28. Emotional functioning/wellbeing | PRO |
| Wound infection | 21 | 11 | 12. Infection and infestation outcomes | Complication |
| Need for re-intervention esthetic correction | 20 | 0 | 36. Need for further intervention | Other |
| Infection (not further specified) | 19 | 1 | 12. Infection and infestation outcomes | Complication |
| Number of complications | 19 | 0 | 38. Adverse events | Complication |
| Erogenous sensibility in neo-phallus | 18 | 4 | 25B. Sexual functioning | PRO |
| Need for re-intervention hematoma | 18 | 4 | 36. Need for further intervention | Re-intervention for complication |
| Satisfaction with sexual function | 17 | 16 | 25B. Sexual functioning | PRO |
| Wound dehiscence | 16 | 4 | 23. Skin and subcutaneous tissue outcomes | Complication |
| Need for re-intervention partial flap loss | 16 | 3 | 36. Need for further intervention | Re-intervention for complication |
| Need for secondary phalloplasty | 15 | 0 | 36. Need for further intervention | Re-intervention for complication |
| Satisfaction with erogenous sensibility in neo-phallus | 15 | 11 | 25B. Sexual functioning | PRO |
| Satisfaction with sex life | 15 | 12 | 25B. Sexual functioning | PRO |
| Delayed wound healing | 14 | 2 | 23. Skin and subcutaneous tissue outcomes | Complication |
| Feelings of masculinity | 13 | 13 | 28. Emotional functioning/wellbeing | PRO |
| Need for re-intervention urologic complication (not further specified) | 13 | 5 | 36. Need for further intervention | Re-intervention for complication |
| Sexual arousal frequency | 13 | 13 | 25B. Sexual functioning | PRO |
| Ability to achieve orgasm | 12 | 9 | 25B. Sexual functioning | PRO |
| Number of Clavien-Dindo graded complications | 12 | 12 | 38. Adverse events | Complication |
| Voiding frequency | 12 | 7 | 19. Renal and urinary outcomes | PRO |
| Ability to achieve orgasm during sexual intercourse with partner | 11 | 10 | 25B. Sexual functioning | PRO |
| Emotional well-being | 11 | 8 | 28. Emotional functioning/wellbeing | PRO |
| Masturbation frequency | 11 | 11 | 25B. Sexual functioning | PRO |
| Need for re-intervention skin graft | 11 | 1 | 36. Need for further intervention | Re-intervention for complication |
| Satisfaction with erection | 11 | 11 | 25B. Sexual functioning | PRO |
| Sexually active | 11 | 8 | 25B. Sexual functioning | PRO |
| Venous flap thrombosis | 11 | 0 | 24. Vascular outcomes | Complication |
| Postoperative bleeding | 10 | 1 | 24. Vascular outcomes | Complication |
| Satisfaction with tactile sensation neo-phallus | 10 | 9 | 20. Reproductive system and breast outcomes | PRO |
| Would recommend surgery to others | 10 | 10 | 32. Delivery of care | PRO |
| Urinary tract infection | 10 | 5 | 19. Renal and urinary outcomes | Complication |
| Urinary voiding symptoms: IPSS mean score | 10 | 10 | 19. Renal and urinary outcomes | PRO |
